# Structural connectivity of the fore- and mid-brain in prairie voles

**DOI:** 10.1016/j.isci.2025.112065

**Published:** 2025-02-20

**Authors:** Kyle R. Gossman, Emalee Andrews, Ben Dykstra, Kyle Ta, Arian Ashourvan, Adam S. Smith

**Affiliations:** 1 Department of Pharmacsology and Toxicology, University of Kansas, Lawrence, KS, USA; 2 Department of Psychology, University of Kansas, Lawrence KS, USA; 3 Program in Neuroscience, University of Kansas, Lawrence KS, USA

**Keywords:** Rodent behavior, Neuroscience, Behavioral neuroscience

## Abstract

Mammals live in complex social systems that require higher order cognition to process and display complex social behaviors. It is suggested that brain networks, such as the social decision-making network (SDMN), have evolved to process such information. Recent functional connectivity studies of the SDMN have revealed distinct network dynamics during different social events across several species. However, the structural mapping of this network is incomplete which limits structural-functional modeling. Here, we assess the structural connectivity of an extended SDMN as well as the fore- and mid-brain afferent projections with the use of cholera toxin subunit-B retrograde tracers and the prairie vole (*Microtus ochrogaster*), a socially monogamous rodent that displays complex social behaviors. This work greatly expands upon the limited structural connectivity of the vole social brain and highlights important regions within the SDMN and other highly innervated regions that may serve as information hubs.

## Highlights

• Many social decision-making network brain regions share bi-directional innervation
• The hub analysis of this network suggests that the anterior hypothalamus is the hub
• Cartographic modeling classifies the mPOA and PAG as non-connector hubs
• The Ent and LH are regions outside of the network with high network connectivity

## Graphical abstract

**Figure:**
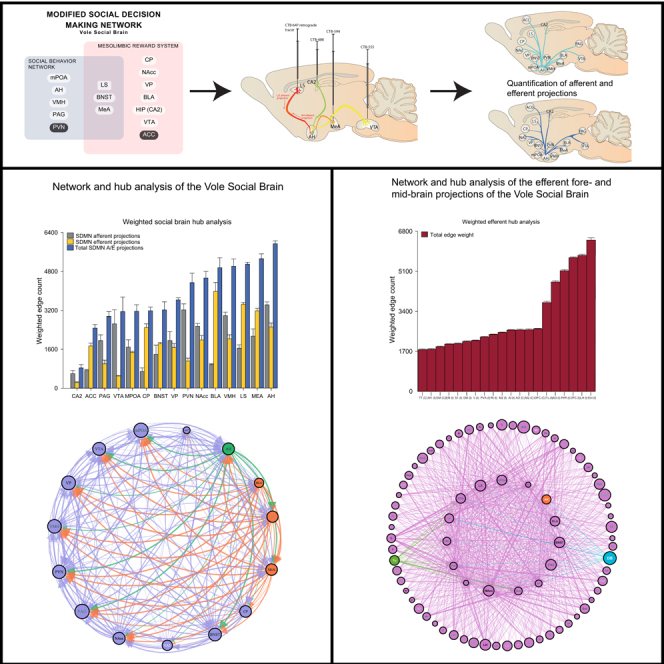

## Introduction

Most mammalian species live in complex social systems. The social brain hypothesis suggests that multiple species have developed larger brains and neural networks to help adapt to increasingly complex social systems and societies, process relevant social information, and produce context-appropriate social behaviors.^1^ This phenomenon is robustly on display in pair bonding species (i.e., socially monogamous systems) which have the largest brains.^2^ The development of larger brains in socially monogamous mating systems may support the complex social behaviors needed to form and maintain such relationships as compared to other mating systems. Although many mating systems share certain behaviors such as aggression, species that are in a socially monogamous mating system display coordinated and synchronized behavior, bi-parental care, occupy and defend common territory, partner-directed affiliation, and selective stranger aggression.^3^ A neural network suggested to have evolved within vertebrates to process and display complex social behaviors is the social decision-making network (SDMN).^4^ The SDMN hypothesis suggests that social behaviors are a reflection of the coactivity of the regions across the entire network rather than the activity of any single region, and this network is thought to be involved in multiple cognitive processes including social attention and motivation, recognition and memory, social communication and emotionality and affective state.^5^^,^^6^^,^^7^ This hypothetical network is thought to be an evolutionarily conserved network, comprised of thirteen regions that form two interconnected circuits: the social behavior network and the mesolimbic reward system.^4^ The social behavior network, first described in 1999, is thought to consist of seven regions, including the anterior hypothalamus (AH), bed nucleus of the stria terminalis (BNST), lateral septum (LS), medial amygdala (MeA), medial preoptic area (mPOA), periaqueductal gray (PAG), and ventromedial hypothalamus (VMH), and is suggested to regulate various aggressive, sexual, and parental behaviors through sex steroid hormones.^8^ The mesolimbic reward system is thought to be comprised of eight regions including the basolateral amygdala (BLA), BNST, caudate putamen (CP), hippocampus (HIP), LS, nucleus accumbens (NAcc), ventral pallidum (VP), and the ventral tegmental area (VTA).^4^ The mesolimbic reward system regulates behaviors such as generating motivation to seek reward, facilitate reinforcement, social choice and decision-making, and valence/salience to cues associated with such outcomes.^9^^,^^10^^,^^11^^,^^12^ Although, this network is thought to be an evolutionarily conserved network, little is known about how this network processes and communicates information for behavioral outputs.

To elucidate the function of this network, the structural-functional relationship needs to be understood at a micro- and macroscale.^13^ Structural connectivity refers to the physical architecture and connections of the brain, while functional connectivity/specialization refers to the activity patterns across the network and how intra- and interregional activity influences context specific behaviors. Although in many rodent models, specific regions and pathways of this network have been studied for their influence of various social and non-social behaviors, the structural-functional relationship of this network in its entirety has limited research, particularly in a rodent model that displays specific social behaviors associated to social attachment or relationship commitment. The prairie vole (*Microtus ochrogaster*) is socially monogamous rodents that form long-term pair bonds between breeding pairs. When these pair bonds have been established, prairie voles will demonstrate well-defined commitment signals, including partner-directed affiliation and selective stranger aggression, which has been well-characterized over the last three decades.^14^^,^^15^ Many of the regions within the SDMN have been shown to regulate various affiliative and aggressive behaviors of prairie voles, but limited research has examined the structural-functional connectivity of the SDMN as a whole.

**Figure 1:**
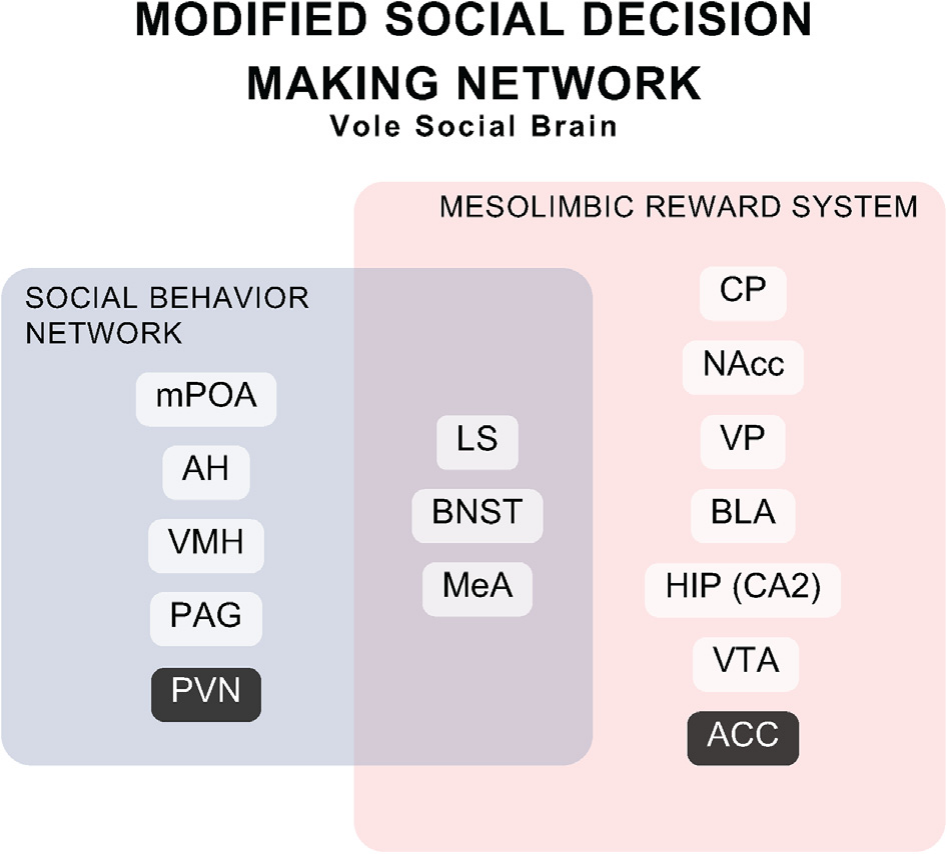
Modified SDMN or vole social brain The anterior cingulate cortex (ACC) and paraventricular nucleus of the hypothalamus (PVN) nodes are different shades to denote the addition to the originally theorized social decision-making network: social behavior network (light blue), mesolimbic reward system (beige), and the three regions in the middle that are shared between the two networks (lateral septum [LS], bed nucleus of the stria terminalis [BNST], and medial amygdala [MeA], [purple]).

Specifically, there is limited research on the structural connectivity, or the physical connections of these regions for the prairie vole brain.^16^^,^^17^^,^^18^^,^^19^^,^^20^ Based on the current studies of social behavior, the structural connectivity, and our previous study of the functional connectivity within this model, we determined that the SDMN should be expanded to include the anterior cingulate cortex (ACC) and paraventricular nucleus of the hypothalamus (PVN).^14^^,^^19^^,^^21^^,^^22^^,^^23^^,^^24^ Although there are more regions that have been shown to regulate social behaviors and should be considered, we have started to build the *vole social brain* with these additions (Figure 1). The purpose of the present study was to (1) assess the structural connectivity of the regions associated with the vole social brain and (2) expand the neuroanatomy of these regions and the connectivity with regions in the fore- and mid-brain of the prairie vole. Here, we used cholera toxin subunit-B (CTB) retrograde tracers and injected the tracer into each of the fifteen regions, where we assessed all the fore- and mid-brain afferent projections to these regions. Finally, we used graph theory to model the structural connectivity of our theoretical network and classify each brain region and reflect on their potential importance.

## Results

### Determination and validation of site injection and tool development

**Figure 2:**
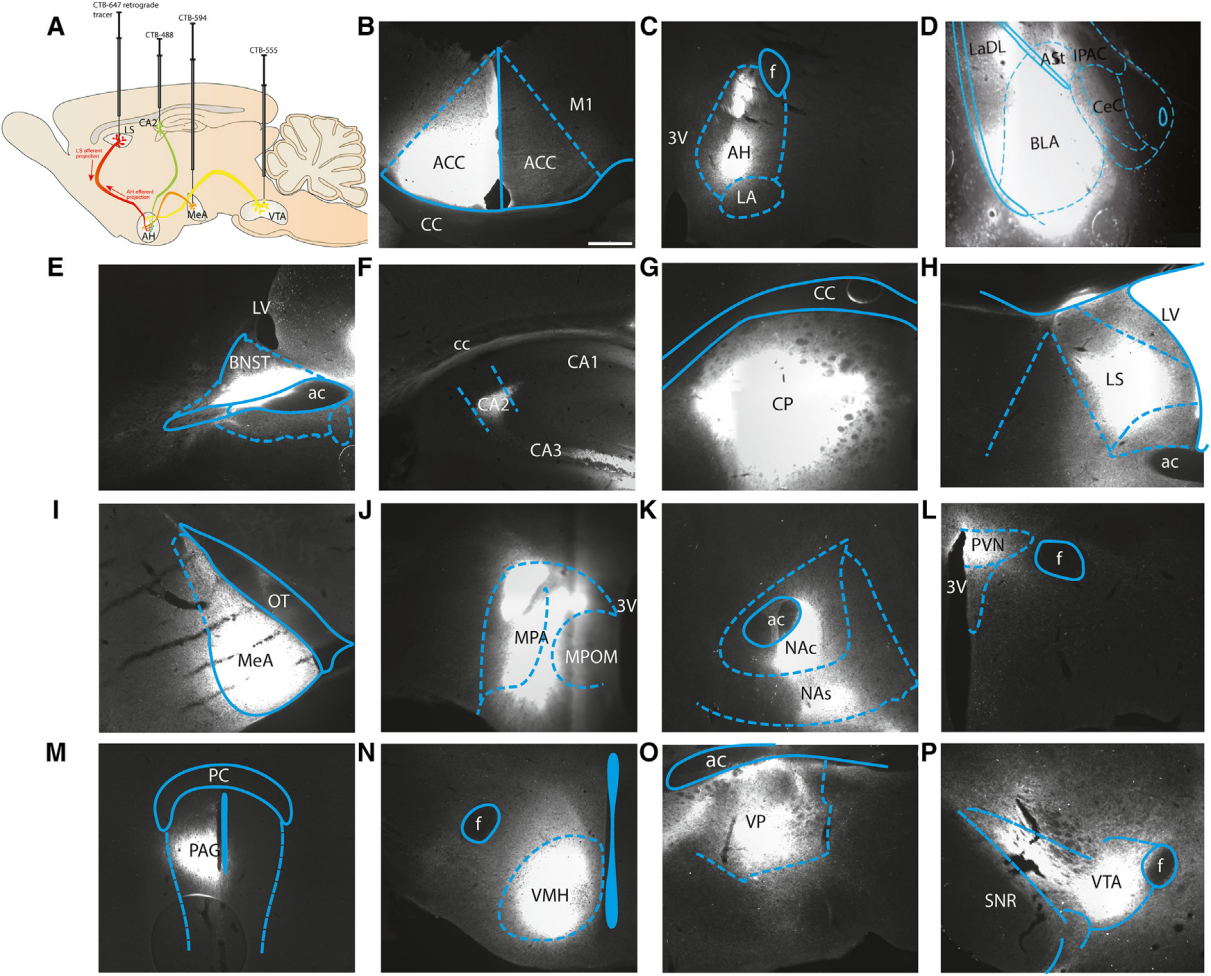
Schematic of injections and visualization of afferent and efferent projection (A) Injection site validation for each of the fifteen regions: anterior cingulate (ACC) (B), anterior hypothalamus (AH) (C), basolateral amygdala (BLA) (D), bed nucleus of the stria terminalis (BNST) (E), CA2 nucleus of the hippocampus (CA2) (F), caudate putamen (CP) (G), lateral septum (LS) (H), medial amygdala (MeA) (I), medial preoptic area (mPOA) (J), nucleus accumbens NAcc (K), paraventricular nucleus of the hypothalamus (PVN) (L), periaqueductal gray (PAG) (M), ventromedial hypothalamus (VMH) (N), ventral pallidum (VP) (O), and ventral tegmental area (VTA) (P). Scale bar, 100 μm.

Compared to other rodent models, the prairie vole model lacks extensive neuroanatomical tracing knowledge, in part, due to limited available tools, such as transgenic lines, viral vectors, and, importantly, a comprehensive vole brain atlas. With the limitations of tracing techniques available for prairie voles, we used cholera toxin subunit-B retrograde tracers (CTB) conjugated to one of four AlexaFluors (AF): 488nm, 555nm, 594nm, or 647nm (CTB-488, CTB-555, CTB-594, and CTB-647), a commonly used tracers in rodents.^25^^,^^26^ We unilaterally injected each CTB-AF into four regions within each subject (Figure 2A). Regions-CTB pairs were randomly assigned. Post-CTB injections, we validated our injection sites of each region (Figures 2B–2P). With respect to the HIP, we specifically targeted the CA2 nucleus as it has been shown to regulate specific social behaviors.^27^ Once we validated our injection sites, we needed a whole-slice image quantification process.

**Figure 3:**
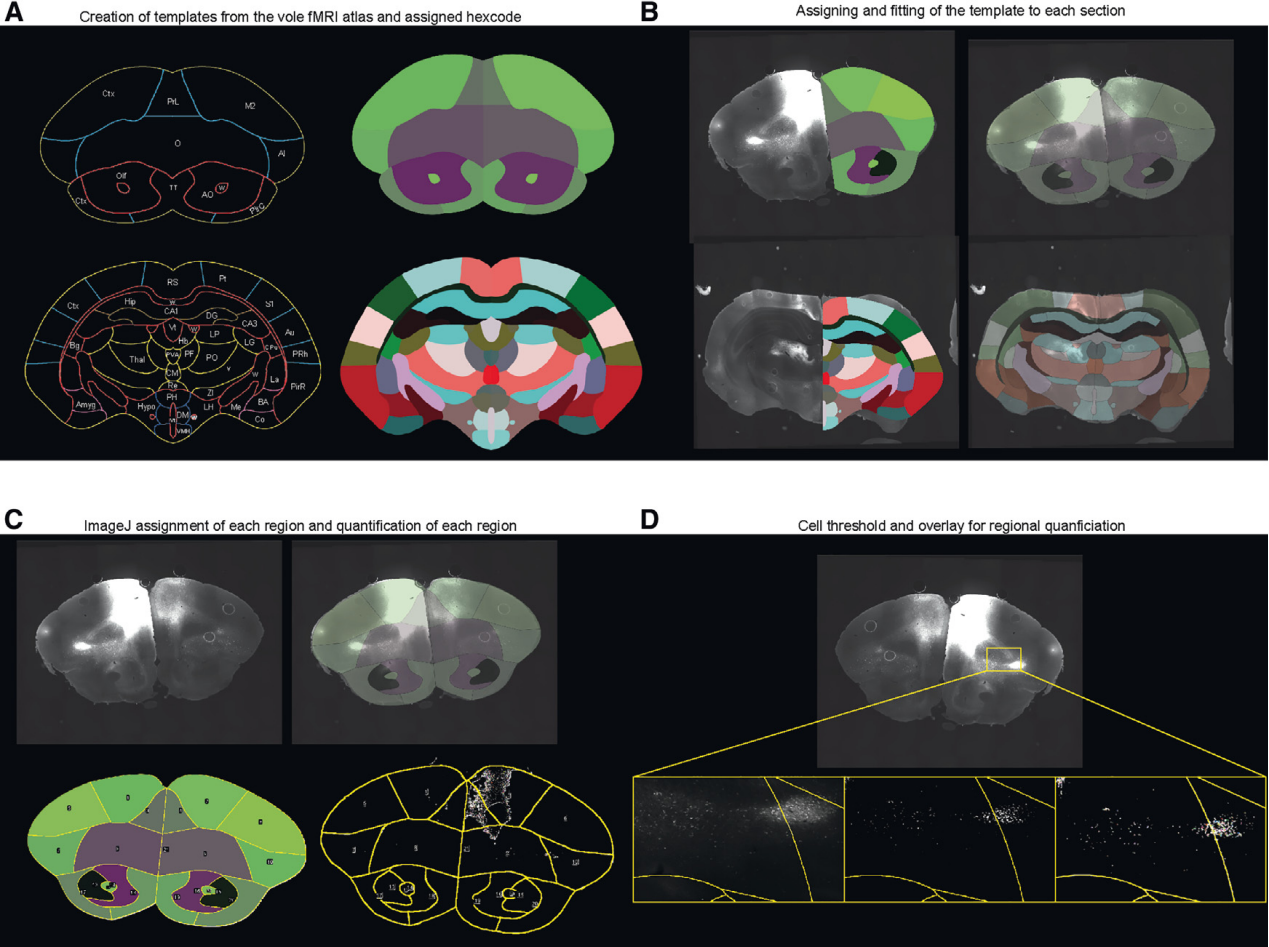
Representative images of the creation and workflow of the tool development and quantification process (A) Demonstrates the annotated vole fMRI atlas (left) and the templates created for each region. (B) Selection and fitting of the template to the image. (C) Representative images of the regional selection by ImageJ (bottom left) and the quantified regions with the template outline for each region (bottom right). (D) Representative images, from left to right, of the signal from the slice, cell signal with a set threshold within ImageJ, and the overlay of the cells quantified.

There is a limited fMRI prairie vole brain atlas, which has been used for autoradiography analysis of oxytocin during various paradigms.^28^^,^^29^ This fMRI vole brain atlas has yet to be implemented into automated template fitting and whole-brain quantification software. With this limitation, we developed a semi-automated template fitting and quantification analysis pipeline to analyze the fore- and mid-brain. Briefly, we modified the fMRI templates by assigning each region, ipsilaterally and contralaterally, a different hex color and converting these templates to vector templates (Figure 3A). The conversion to vector templates would allow for the adjusting of the overall template to fit the whole-slice and to adjust each region specifically for a proper fit (Figure 3B). For the fore- and mid-brain specifically, 35 templates were created from the fMRI vole brain atlas and modified when needed for use (Figures S1A and S1B). Once the templates were properly fitted in Illustrator (Adobe Creative Cloud: Adobe Illustrator), the template and image could be imported into ImageJ where we wrote a macro to automate the process and allow ImageJ to read each hexcode to register and label each region before generating cell segmentation and counts for each region, ipsilateral and contralateral (Figures 3C and 3D). This tool development allows for one of the first vole based semi-automated template fitting and quantification processes.

### Vole social brain connectome of the prairie vole fore- and mid-brain

**Figure 4:**
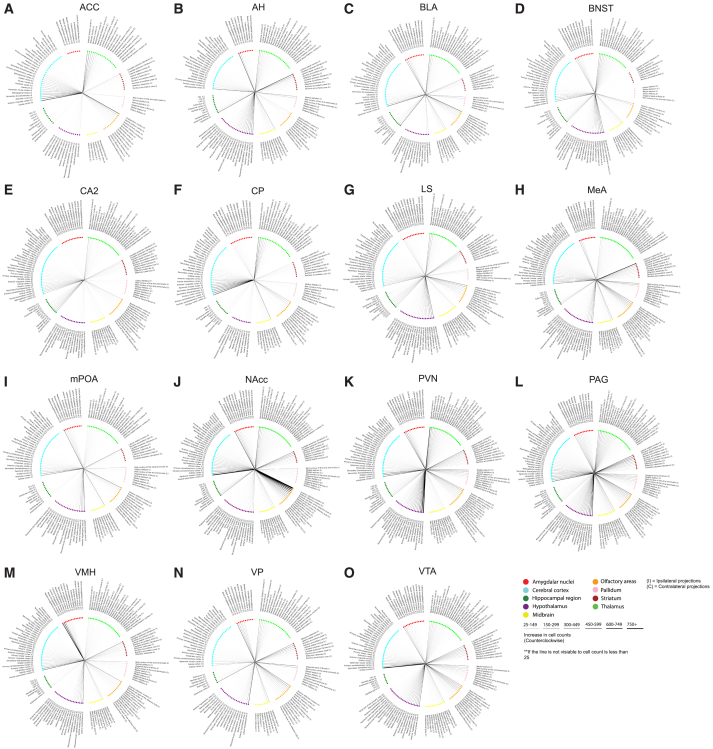
Radial graphs summarize the cell counts for the fore- and mid-brain efferent projections to the regions associated with the vole social brain (A) Anterior cingulate cortex (ACC). (B) Anterior hypothalamus (AH). (C) Basolateral amygdala (BLA). (D) Bed nucleus of the stria terminalis (BNST). (E) CA2 nucleus of the hippocampus (CA2). (F) Caudate putamen (CP). (G) Lateral septum (LS). (H) Medial amygdala (MeA). (I) Medial preoptic area (mPOA). (J) Nucleus accumbens (NAc). (K) Paraventricular nucleus of the hypothalamus (PVN). (L) Periaqueductal gray (PAG). (M) Ventromedial hypothalamus (VMH). (N) Ventral pallidum (VP). (O) Ventral tegmental area (VTA). The colors of the nodes represent what nuclei a particular region is in, both ipsilateral (I) and contralateral (C). Bar thickness denotes an increase in cell count within each nucleus, and regions are organized into major areas and arranged from least to greatest innervation. n = 3–5.

The first purpose of this study was to expand upon the structural connectivity of the fore- and mid-brain of prairie voles. As mentioned, there are limited studies examining the neuroanatomical tracing within the vole brain. First, we validated the injection sites of the target regions including: ACC (*n* = 5, Figure 2B), AH (*n* = 4, Figure 2C), BLA (*n* = 4, Figure 2D), BNST (*n* = 3, Figure 2E), CA2 (*n* = 3, Figure 2F), CP (*n* = 5, Figure 2G), LS (*n* = 5, Figure 2H), MeA (*n* = 5, Figure 2I), mPOA (*n* = 4, Figure 2J), NAcc (*n* = 5, Figure 2K), PVN (*n* = 4, Figure 2L), PAG (*n* = 4, Figure 2M), VMH (*n* = 3, Figure 2N), VP (*n* = 5, Figure 2O), and VTA (*n* = 4, Figure 2P). Next, we provide radial graphs of the fore- and mid-brain regions (80 regions total, ipsilateral and contralateral) that project into the regions associated with the vole social brain: ACC (Figure 4A), AH (Figure 4B), BLA (Figure 4C), BNST (Figure 4D), CA2 (Figure 4E), CP (Figure 4F), LS (Figure 4G), MeA (Figure 4H), mPOA (Figure 4I), NAcc (Figure 4J), PVN (Figure 4K), PAG (Figure 4L), VMH (Figure 4M), VP (Figure 4N), and VTA (Figure 4O). All quantified images can be found on the open-source repository DABI: https://doi.org/10.18120/t6yj-dg76. Certain regions may have lower cell counts as displayed within the images, as the CTB label was too intense for accurate cell quantification. However, these radial graphs summarize all the fore- and mid-brain regions that project to each of the 15 regions. This greatly expands our knowledge of the structural connectivity of the vole brain.

### Most regions in the vole social brain share bi-directional projections

**Figure 5:**
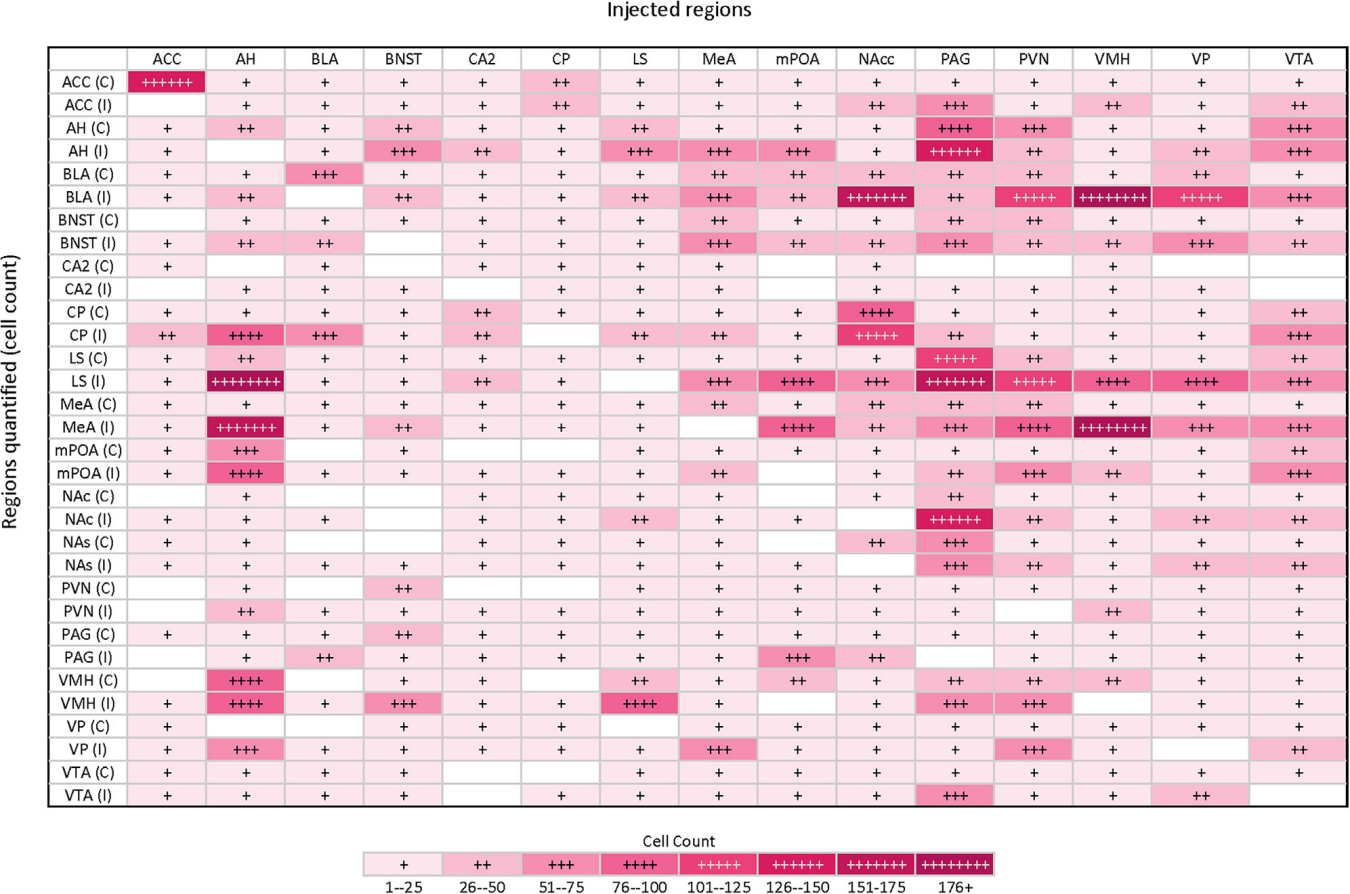
The regions associated with the vole social brain demonstrate high innervation with one another, as each region shares physical projections with many if not all of the other regions This graph represents the injected regions and regional afferent ipsilateral (I) and contralateral (C) projections. Increase in cell count is displayed by increase in the number of “+” and darkness of color. Unpaired T-Test between afferent and efferent ipsilateral, contralateral, and total cell counts (∗*p* < 0.05). *n* = 3.

Beyond expanding upon the structural connectivity fore- and mid-brain within the prairie voles, we wanted to further characterize the theorized vole social brain. As mentioned, this network consists of 15 regions suggested to be involved in the regulation of social behaviors. For this theoretical network to exist, it is suggested that all regions within the network need to share at least one physical projection with another region associated with the network.^30^ With the use of the CTB retrograde tracers, the ipsilateral and contralateral afferent projections, or regions that project into the injected region, and the ipsilateral and contralateral efferent projections, or the projection each injected region projects to, can be established for the regions within this network. Unpaired t tests demonstrate a significant difference between ipsilateral and contralateral projections (*p* < 0.001). Of the possible 210 projection combinations between the 15 regions within the vole social brain, our structural connectivity data of these regions demonstrates that there are 5 regions that do not share projections, 3 that are unidirectional, and 202 are bi-directional projections, however, some regions within this network share sparse projections (Figure 5). This suggests that additional regions may need to be added to this network. To note, certain regions, such as those within the hypothalamus have lower cell counts as displayed within the images, as the CTB label was too intense for accurate cell quantification.

### Hub analysis suggests that the anterior hypothalamus is a hub of the vole social brain

**Figure 6:**
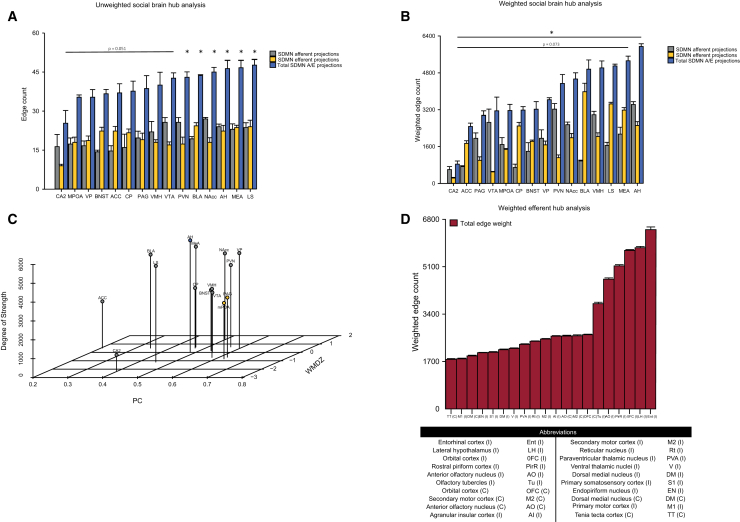
The unweighted hub analysis for the regions within the vole social brain suggest that there is no hub of this network (A) The weighted hub analysis for the regions within the vole social brain suggests that the anterior hypothalamus (AH) has the highest degree of connectivity. (B) Cartographic 3D scatterplot, which graphs the degree of strength (total of cell count), participation coefficient (PC), and within-module degree *Z* score (WMDZ), to further classify the non-hub regions. (C) The medial preoptic area (mPOA) and periaqueductal gray (PAG) are classified as non-connector hubs (yellow circles), while all other regions are classified as peripheral hubs (gray circles). The anterior hypothalamus (AH) is colored red to represent our hub. A weighted hub analysis for efferent projections of regions in the fore- and mid-brain indicates that the lateral hypothalamus (LH) followed by the orbital cortex (OFC) demonstrates the highest connectivity with the regions of the vole social brain along with the abbreviations for regions of the fore- and mid-brain shown. Presented from highest cell count to lowest cell count in the table. (D) Edge count refers to whether a region has a projection or not, regardless of the number of quantified cells. Weighted edge count refers to the addition of the weight or cell count +/− SEM of all edges or projections to one region. One-way ANOVA with Bonferroni post-hoc comparisons (*p* < 0.05). *n* = 3.

To further characterize this network, we used a graph theory approach to examine if there is a mathematic hub or a region with a high degree of connectivity within the network.^30^ The hub of a network is thus defined as a crucial region for the network for efficient communication.^30^ There are two ways, unweighted and weighted, to assess a potential hub(s) of a network. An unweighted hub analysis is calculated by the nodal degree or counting the number of edges connected to each node.^31^ We calculated the individual and total ipsilateral and contralateral afferent and efferent projections of the regions within the vole social brain. After assessment of the total afferent and efferent projections, the LS, MeA, and AH demonstrate the highest degree of connectivity, but this does not appear to be distinct as eight other regions have one to two less total afferent/efferent projections (Figure 6A). The CA2 demonstrates the lowest degree of connectivity and has significantly less physical projections than the top six regions (LS, MeA, AH, NAcc, BLA, and PVN; F_(14,30)_ = 3.515, *p* < 0.001) The CA2, mPOA, and VP demonstrate the lowest degree of connectivity within the network.

**Figure 7:**
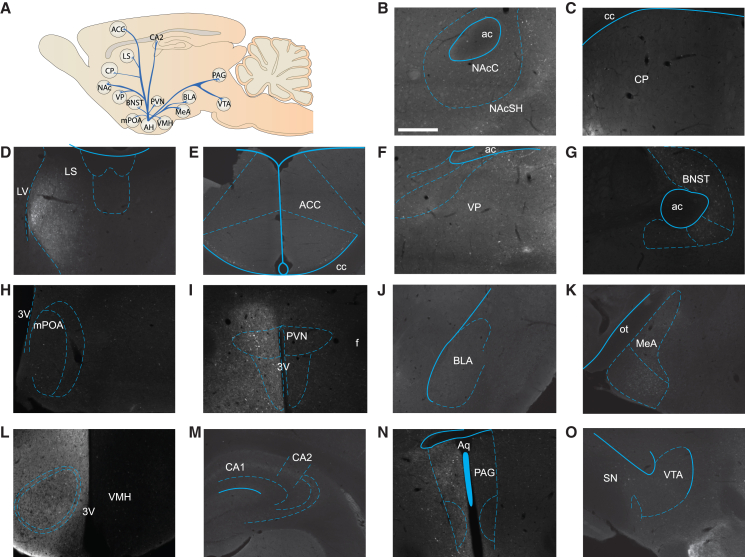
Schematic of afferent projections of the AH (A) Representative images of the afferent projections of the vole social brain to the anterior hypothalamus (AH): nucleus accumbens (NAc) (B), caudate putamen (CP) (C), lateral septum (LS) (D), anterior cingulate (ACC) (E), ventral pallidum (VP) (F), bed nucleus of the stria terminalis (BNST) (G), medial preoptic area (mPOA) (H), paraventricular nucleus of the hypothalamus (PVN) (I), basolateral amygdala (BLA) (J), medial amygdala (MeA) (K), ventromedial hypothalamus (VMH) (L), CA2 nucleus of the hippocampus (CA2) (M), periaqueductal grey (PAG) (N), ventral tegmental area (VTA) (O). The brightness/contrast of these images have been increased uniformly for visual purposes. Scale bar, 100 μm.

**Figure 8:**
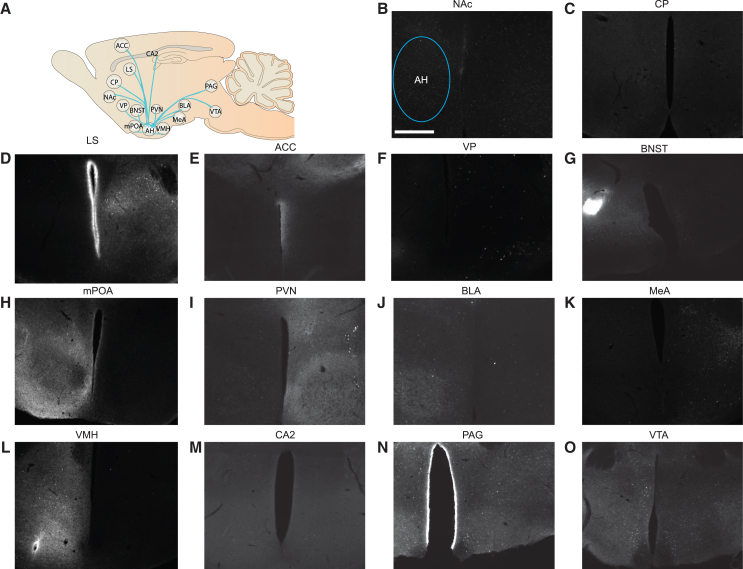
Schematic of efferent projections of the AH (A) Representative images of the efferent projections of the anterior hypothalamus (AH) to the regions of the vole social brain: nucleus accumbens (NAc) (B), caudate putamen (CP) (C), lateral septum (LS) (D), anterior cingulate (ACC) (E), ventral pallidum (VP) (F), bed nucleus of the stria terminalis (BNST) (G), medial preoptic area (mPOA) (H), paraventricular nucleus of the hypothalamus (PVN) (I), basolateral amygdala (BLA) (J), medial amygdala (MeA) (K), ventromedial hypothalamus (VMH) (L), CA2 nucleus of the hippocampus (CA2) (M), periaqueductal gray (PAG) (N), ventral tegmental area (VTA) (O). The brightness/contrast of these images have been increased uniformly for visual purposes. Scale bar, 100 μm.

The weighted hub analysis assigns a “weight” (e.g., cell count or cell density) to the edge or connection between regions, and those weighted edges can be summed to determine a potential hub. Here, we assigned the number of cells labeled by the CTB tracers to assign a weight to each edge between regions. Our weighted hub analysis found that the AH demonstrates the highest level of connectivity (Figures 6B, 7A–7O, and 8A–8O), which is statistically greater than the CA2 (F_(14,30)_ = 2.537, *p* = 0.016). The MeA has the second highest connectivity compared to the other regions. The CA2 demonstrated the lowest degree of connectivity followed by the ACC. To further classify the other regions outside of the AH, we used cartographic network modeling.^32^ Cartographic network modeling uses the within-module degree *Z* score (WMDz) or how connected one node is to other nodes within its module, the participation coefficient (PC) or how connected one node is with other regions outside its module, and the degree of strength or total cell count. A module refers to a cluster of regions determined by a dendrogram or the calculation and assessment of similar structural projection patterns of one region to a group of regions. To calculate the WMDz and PC we used the edge weight, or the total cell count of a projection from one region to another, and the total weight used in the hub analysis was used for the degree of strength. The mPOA and PAG are classified as non-hub connectors or regions that have at least two edges within their module and then a PC < 0.80 (Figure 6C). This suggests that these regions are well connected with other regions, however, the high connectivity for a connector hub is not met. Beyond these two regions and the AH, all other regions are classified as non-hub peripheral nodes or regions that demonstrate at least 60% of their edges within their module. Lastly, the AH is classified as our hub of this network; however, since the AH is in a module by itself, the WMDz will always equal 0. Lastly, we wanted to assess if there was a potential efferent hub of regions outside of the regions associated with the vole social brain. Here, we present the twenty regions with the highest connectivity of efferent projections to regions within the vole social brain. The weighted hub analysis suggests that the entorhinal cortex (Ent) is highly interconnected with the regions of the vole social brain, followed by the lateral hypothalamus (LH) and the orbital cortex (OFC) (Figure 6D). Together, the weighted hub analysis data indicates that the AH is a structural hub for this network and that the Ent, LH, and OFC are heavily connected with regions of the vole social brain and may warrant inclusion into vole social brain model.

### Our vole social brain displays distinct modularity compared to the theorized SDMN

To further examine and compare our vole social brain network with the theorized SDMN, we used graphical and mathematical modeling, refer to the methods section for specific calculations and terms. To identify the densely connected modules we first used hierarchical clustering or generated dendrograms to observe modular organization or clusters of regions that share similar distributions of afferent and efferent projection counts (dendrograms were cut such that it represented three modules as the SDMN^14^^,^^33^). After identifying the modules, we next created our edges. To do this, we assigned a value between 0 and 0.9 based on the weight of the edge to demonstrate connections between regions. We also calculated a PC or the strength of inter-modular connectivity and a modified within-module degree *Z* score or the strength of intra-modular connectivity for all regions within the network.

**Figure 9:**
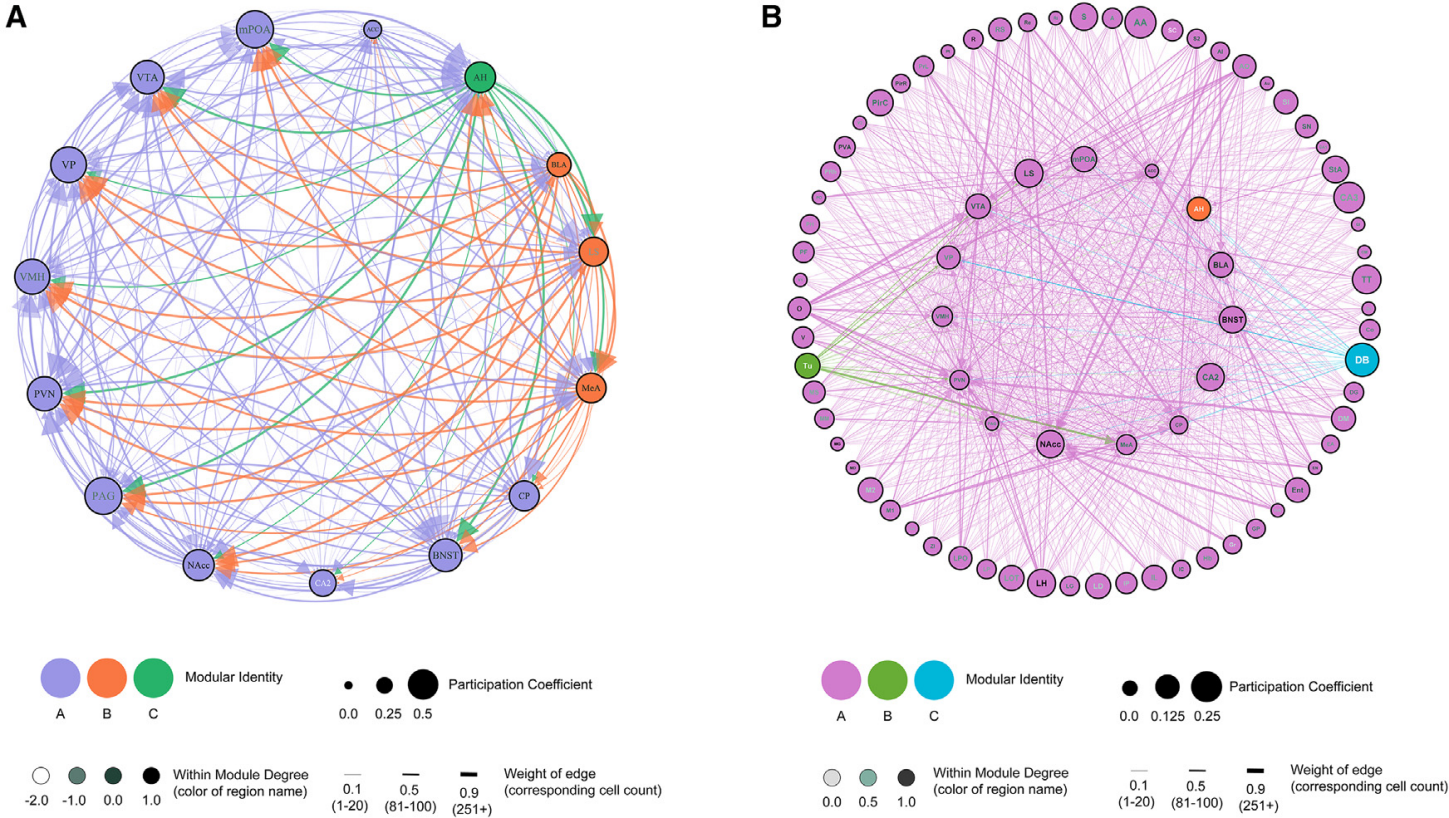
Structural connectivity networks (A and B) Regions associated with the vole social brain (A), and all fore- and mid-brain regions quantified (B). Nodes/brain regions in each network are represented by circles. The color of the node represents the modular identity (regions with the same colors are in the same module). The shade of text within the circle represents the within-module degree *Z* score (WMDz) (the darker the color = higher score). The size of the node/circle represents the participation coefficient (PC) (larger the circle = higher the PC). The thickness of the line or edges and size of the arrow between nodes represents the weight of the projection (thin = lower weight, thick = higher weight). The arrow demonstrates the direction of the projection, and the color of the arrow is correlated with the node where that projection originates from.

Using these calculations, we were able to generate structural connectivity models to better visualize the connections between regions and the networks (Figure 9A). Similar to the theorized SDMN, our model displays three clusters or modules (Figure S2A), however, the regions within each cluster differ from the theorized clusters. The first cluster is made up of 11 regions including: ACC, BNST, CA2, CP, mPOA, NAcc, PAG, PVN, VMH, VP, and VTA. The second cluster consists of the BLA, LS, and MeA. Lastly, the AH is an “isolate” within our network model, which is also the calculated hub. This suggests that the AH may be a key connector hub or a hub that disseminates the majority of the necessary information throughout this network. We also modeled the projections of all the other regions quantified outside the VSB (Figures 9B and S2B). Our model displays the VSB regions in the inner-circle and the regions that cluster together in the outer-circles. This allows for visualization of other highly connected regions into the VSB, which could suggest specific regions such as the entorhinal cortex (Ent), orbital cortex (OFC), and LH, to be incorporated into the VSB in future studies.

## Discussion

The social brain hypothesis suggests that circuits and networks evolved to process information perceived from a complex social society.^1^ Understanding neural networks is crucial as the cognitive processing of socially relevant information and output of social behaviors is based on the shared activity of interconnected brain regions rather than one single region.^4^^,^^34^ The SDMN is suggested to be a cluster of brain regions involved in the processing of social cues and the display of complex, context-appropriate behaviors throughout the various social environments and encounters an individual may experience.^4^ However, a comprehensive mapping of the efferent and afferent projections between each brain region of this network has yet to be completed. The present study used naive male and female prairie voles and CTB retrograde tracers to assess the structural connectivity of the SDMN, with the incorporation of the ACC and PVN as two regions shown to be involved in social behaviors.^19^^,^^23^ With the development of a novel tool for the prairie vole model, which used semi-automated template fitting and whole-slice quantification, we expanded upon the neuroanatomical fore- and mid-brain ipsilateral and contralateral afferent and efferent projections of the fifteen regions within the vole social brain.

As the SDMN is suggested to be a highly conserved network among vertebrates, a variety of species have been used to study the functional characteristics of this network, including voles (cFos during social interaction),^14^ fish (immediate-early genes during social interaction),^35^^,^^36^ anole (catecholamines during reproduction and aggression),^37^ and rats (receptor binding and distribution).^38^^,^^39^ The functional connectivity of regions across the network is influenced by the structure of the network.^40^^,^^41^ Specifically, activity between regions is dependent, in part, on whether regions share direct or indirect connections as well as the density and directionality of those connections. Furthermore, the role of a brain region in network dynamics is dependent, in part, on its structural integration with regions in that network. Surprisingly, the structural connectivity of the regions that make up the SDMN has yet to be assessed in its entirety. Therefore, the first goal of this study was to significantly expand upon the mapping of structural connectivity in the SDMN, so we assessed the contralateral and ipsilateral afferent and efferent projections of each region. The regions associated with the SDMN in the vole brain demonstrated a first-order network, meaning a significant number of regions shared direct projections to a majority of the other regions.^42^ Although many of these regions in this network share bidirectional projections, the projection density varied greatly from sparse to dense. For example, although the CA2 nucleus of the HIP demonstrates direct projections to other regions examined, the density of such projects was sparse. This is similar to the mouse brain where the CA2 demonstrates moderate to high innervation to only a few regions including the PVN, medial septum, supramammillary nucleus, and other HIP nuclei.^43^ This could indicate that other regions need to be incorporated into the SDMN, as the CA2 is part of an interconnected circuit with the entorhinal cortex, subiculum, and other sub-nuclei of the HIP, including the CA1, CA3, and dentate gyrus.^44^ To that end, we examined the structural projections of the fore- and mid-brain to the regions of the SDMN (presented later in our Discussion), and with that information, we can speculate about other regions that could be incorporated into this network. Furthermore, these structural connectivity data can be used to predict aspects of functional relationships between regions, including directionality and direct vs. indirect signaling. Further structural-functional analysis should be done to examine how this network works as a collective to regulate social behaviors, as well as examine other potential regions to be involved in the vole social brain.

Second, we wanted to determine potential hubs or regions with a high degree of structural connectivity throughout the network. Our structural connectivity hub analysis, which indicated the AH as a potential overall hub of this network, displays a similar result with previous functional connectivity work of a prosocial network at resting-state.^45^ If the AH indeed acts at a hub, the AH would work to process and disseminate information throughout the network. Particularly, in pair-bonded male prairie voles, the AH has been shown to regulate both partner-directed affiliation and stranger-directed aggression and is active during both types of encounters in bonded males.^20^^,^^46^^,^^47^ Briefly, vasopressin and corticotrophin-releasing factor signaling in the AH promote stranger aggression while 5-HT signaling elicits partner affiliation. Moreover, these neurochemical inputs can promote either affiliation or aggression and can inhibit signaling for opposing behavioral actions, which may support the expression of context-appropriate social behaviors.^20^ The fact that the AH is also heavily integrated into the SDMN, both through afferent and efferent projections, suggests that inputs that selectively modulate AH neural activity may also be able to regulate the functional output of the SDMN at large. In other animal models, the AH is involved in other social behaviors such as approach and avoidance toward conspecifics, stress response, predator defense mechanisms, and conspecific aggression.^48^^,^^49^^,^^50^^,^^51^ Therefore, further research should focus on the role of the AH in modulating actions of the SDMN overall and the promotion of context-appropriate social behaviors across various social encounters. Based on our weighted hub analysis, the MeA may also act as a hub. The MeA poses as an interesting region as it is suggested to regulate social approach-avoidance, mating behaviors, aggression, and other general social affiliative behaviors in prairie voles and other species as well as receive major input for sensory information such as olfactory cues.^52^^,^^53^^,^^54^^,^^55^^,^^56^ Graph theory suggests that hubs can be either provincial hubs, regions heavily connected and relay information within the same module, or connector hubs, regions that are heavily connected and relay information to other modules or networks.^30^ In the theorized SDMN model, the LS, BNST, and MeA serve as potential connector hubs as they demonstrate high innervation between both the social behavior network and the mesolimbic reward system, while the AH is not theorized as a hub. However, based on our clustering analysis, the AH and MeA could each act as connector hubs between the social behavior network and mesolimbic reward system.

Although a weighted hub analysis is a traditional way to reveal hubs, we wanted to further classify the other 14 regions, outside the AH, as potential non-hub and hub regions, we used cartographic modeling which plots the PC and the within-module degree *Z* score.^32^^,^^57^ Unlike our weighted hub analysis that suggested the MeA to be another hub along with the AH, all regions were classified as peripheral nodes beside the mPOA and PAG, which were classified as non-hub connectors. Non-connector hubs are regions that are suggested to have higher connections with regions outside its module than within.^32^ Interestingly, the mPOA and PAG are within the same module, which may suggest that one region serves as an input region to its module and the other sends this information out to other modules. Based on the afferent and efferent projections of the mPOA and PAG, it may suggest that the mPOA plays a role in receiving and projecting various social behaviors as it receives similar afferent and efferent projections.^58^ As for the PAG, our data demonstrates strong afferent projections, similar to rats, which may process information from regions within the SDMN and then project to a variety of regions within and outside of the SDMN, including many cortical regions, the brainstem, and regions of the thalamus.^59^^,^^60^^,^^61^^,^^62^ Although there is limited research within the vole model, in other rodent models, these two regions have been shown to regulate reward, social play, and aggression.^63^^,^^64^^,^^65^^,^^66^^,^^67^^,^^68^^,^^69^ Peripheral nodes are nodes that have low connectivity to other modules.^32^ As many of our regions are classified as peripheral nodes, this may suggest that these regions serve a specific role in pair bond formation and maintenance, and project this information mainly within their own module. The peripheral nodes then use connector hubs to have this information passed to other regions outside of their modules.^32^ The NAcc fell short of being classified as a connector hub but had high connectivity in the network. This is another interesting region to note as the role of the NAcc in modulating pair bond formation and maintenance in prairie voles has been well established. The NAcc has been well documented as oxytocin and dopamine 2 receptor expression and function are necessary for pair bond formation and dopamine 1 receptor expression is necessary for pair bond maintenance and aggression.^9^^,^^70^^,^^71^^,^^72^ For other rodent species, the NAcc has been shown to regulate many social behaviors including promoting social interaction and play in rats,^73^^,^^74^^,^^75^ social dominance in mice, as well as anxiety-like behaviors,^76^^,^^77^^,^^78^^,^^79^ and regulates social approach and interaction in California mice.^80^^,^^81^ As a potential connector hub, it would suggest that along with the inputs into the NAcc, outputs from the NAcc may be important in a pair bond and social-related context and should be explored in such. However, with the weighted hub analysis and cartographic modeling demonstrating the potential of only one hub and majority of peripheral nodes, this could also suggest that SDMN is too simplistic and more modules or circuits within the SDMN exist or the SDMN is a smaller circuit within a larger network, which would be consistent with our previous functional connectivity modeling.^14^ It also may suggest that a structural hub may not exist, and further exploration into the structural-functional relationship is necessary to establish the roles of each node in this network.

The third purpose of this study was to examine the efferent fore- and mid-brain projections to the vole social brain. The data suggest that the vole social brain is highly innervated with the fore- and mid-brain regions. Although we incorporated the ACC and PVN into the theorized SDMN, literature suggests that other regions regulate social behaviors and are highly innervated with regions within the SDMN and vole social brain (i.e., insular cortex based on structural and functional connectivity^82^). An efferent weighted hub analysis was performed to determine if other regions should be incorporated into this network based on their structural connectivity. We presented the thirty-six regions with the highest degree of connectivity, and the Ent and LH are two regions with the highest degree of connectivity with the other vole social brain regions. These two regions are thought to be highly interconnected across rodent brains and are connected to the regulation of certain social behaviors.^83^ Research thus far has shown that the Ent is heavily connected with the hippocampus, however, our research suggests that the Ent also has major projections throughout the vole social brain. Currently, there are limited studies on how the Ent regulates social behavior. However, it is suggested to regulate social memory and recognition, as well as the regulation of navigating new spaces and individuals.^84^^,^^85^^,^^86^^,^^87^ With social species, particularly monogamous species such as the prairie vole, the Ent may be an important region to process the difference between a partner and a stranger conspecific and display context-appropriate behaviors. The LH is also suggested to be highly interconnected with the regions within the vole social brain and much of the research has provided insight into innate behaviors.^88^^,^^89^ Currently, there are limited studies on how the LH regulates social behavior, with one suggesting that the LH regulates defensive behaviors and another suggesting the LH promotes dominance in competition.^90^^,^^91^ With the high innervation into the social brain, the Ent and LH are both intriguing regions to study further for their roles in social behavior.

Beyond regional analysis, we wanted to model and assess the network based on structural connectivity. Thus far, regions of the SDMN have been studied to map the regional distribution of various receptors or changes in neuronal activity based on various social interactions.^37^^,^^39^^,^^92^ However, only a few studies have examined the functional connectivity of this network, and the structural connectivity has yet to be fully characterized. Without structural maps of efferent and afferent pathways across the network, predictive modeling of network functional connectivity is limited. The studies that have assessed the functional connectivity of the SDMN, including voles, anoles, fish, and humans, all have demonstrated modular identity (i.e., number and regional composition of modules) that varies from the theorized SDMN model.^14^^,^^36^^,^^37^^,^^39^^,^^93^^,^^94^^,^^95^^,^^96^ Although these functional connectivity studies demonstrate varying modular organization, there are similarities in defined hubs, or important regions, within the network between our structural connectivity study and the functional connectivity work. These hubs include the AH and amygdala in voles, anole, and humans.^37^^,^^97^ It is suggested that the amygdala is a multisensory processing region, that heavily projects to the thalamus and hypothalamus for further sensory processing and potential motor output.^98^^,^^99^ fMRI studies in humans have suggested that the thalamus and hypothalamus are potential hubs in the default network, resting state network, and emotional states such as joy and empathy.^95^^,^^100^^,^^101^^,^^102^^,^^103^ Hypothalamic nuclei regulate social reward as well as innate behaviors including sexual, parental, and aggressive behaviors.^69^^,^^104^ Our data demonstrates the high structural integration of hypothalamic and amygdalar nuclei such as the AH and MeA into the SDMN, suggesting both as hubs. Our work also highlights the importance of the overall hypothalamic nuclei (i.e., LH, AH, VMH, PVN, and mPOA), which are all highly connected with each other and most regions of the SDMN. As different vertebrate models have demonstrated varying network models, further research should be conducted to study the structural-functional relationship, as well as studied across vertebrate models to assess how behavioral evolution influences potential hubs and neurocircuitry and processing of multisensory information. Another region suggested to be a hub is the prefrontal cortex (PFC), specifically the ventral portion or the cingulate nucleus of the PFC. For example, one fMRI study noted that the ACC was a consistent hub of default and task stimuli (i.e., emotion, language, motor, and social) suggesting the importance in the maintenance and sustained cognitive processes.^105^^,^^106^ Although other models and studies of the SDMN have not incorporated the PFC within their analysis, the cortical organization (basic elements and pattern of organization) is suggested to be highly conserved among vertebrates.^107^ Our hub analyses did not identify the ACC as a hub of the SDMN in the prairie vole brain. However, the functionality of the PFC is suggested to be more developed in humans as compared to other vertebrate species including the size and volume of subregions within the PFC as well as the processing of information (bottom-up vs. top-down circuity).^107^ Thus, while the SDMN is suggested to be conserved across vertebrates, there may be some evolutionary divergences.

### Limitations of the study

Collectively, this work defines the structural connectivity of the ipsilateral and contralateral afferent and efferent fore- and mid-brain of a modified SDMN or a *vole social brain*. We have expanded upon our limited knowledge of the prairie vole brain architecture while developing a novel analytical tool for this model. However, there are limitations within our study that future research should address. First, this study pools together male and female connections. Future studies should examine potential differences of specific projections and how these projects may alter functional differences. Also, our study outlines general regional projections and although our research details important structural connections within the vole brain, further structural analysis should be done for each target region to assess potential variations of projections based on subnuclei of a region (anterior vs. posterior and medial vs. lateral). Future research should also focus on specific cell populations that project from one region to another. Also, there should be a focus on the development of a more comprehensive vole brain atlas which will allow for an automated template fitting with more defined regional boundaries and quantification process like other rodent models. In addition, it will be meaningful to assess how the structural mapping and the structural-functional relationship of the vole social brain changes following major social life events (i.e., pair bonding and parenthood). Future research should address such limitations as well as promote research into the combination of the structure-function relationship and novel circuits or regions (i.e., MeA, Ent, and LH) during the display of social behaviors. Ultimately, the structural architecture of a neural network is one factor that applies constraints to the functional connectivity of a network. However, research is required to determine if putative mechanisms and functions of a network occur.

## Resource availability

### Lead contact

Further information and requests for resources and reagents should be directed to and will be fulfilled by the lead contact, Adam S. Smith (adamsmith@ku.edu).

### Materials availability

This study did not generate new unique reagents.

### Data and code availability

• Data: Images analyzed for this study have been deposited at DABI (https://doi.org/10.18120/t6yj-dg76).
• Code: This manuscript does not report original code.
• All other items: Any additional information required to reanalyze the data reported in this paper is available from the lead contact upon request.

## Acknowledgments

We would like to thank Sydney Vanmeerhaeghe for her assistance with image analysis and the KU ACU staff for veterinary and husbandry care of the vole colony at KU. We would also like to thank the funding that supported this work, the BRAIN Initiative through the 10.13039/100000002National Institutes of Health (R01-NS113104), the 10.13039/100000025National Institute of Mental Health (R01-MH133123), KU PREP Program through the 10.13039/100000057National Institute of General Medical Sciences (R25-GM078441), and K-INBRE through the 10.13039/100000057National Institute of General Medical Sciences (P20-GM103418).

## Author contributions

K.R.G., K.T., and A.S.S. contributed to the conception and design of the study. K.R.G. and B.D. organized the database. K.R.G., E.A., and B.D. helped with the tissue processing and image analysis. K.R.G., E.A., B.D., A.A., and A.S.S. performed the statistical analysis. K.R.G. wrote the first draft of the manuscript. A.S.S. and A.A. helped write sections of the manuscript. All authors contributed to the manuscript revision, proof reading, and approved the submitted version.

## Declaration of interests

The authors declare that the research was conducted in the absence of any commercial or financial relationship that can be construed as a potential conflict of interest.

## STAR★Methods

### Key resources table

**Table:** 

REAGENT or RESOURCE	SOURCE	IDENTIFIER
**Chemicals, peptides, and recombinant proteins**		

Cholera toxin subunit-B retrograde tracer (CTB) 488	Invitrogen	Lot no. 2155272
Cholera toxin subunit-B retrograde tracer (CTB) 555	Invitrogen	Lot no. 2129666
Cholera toxin subunit-B retrograde tracer (CTB) 594	Invitrogen	Lot no. 2387433
Cholera toxin subunit-B retrograde tracer (CTB) 647	Invitrogen	Lot no. 2155574

**Deposited data**		

Analyzed data	This manuscript	DABI: https://doi.org/10.18120/t6yj-dg76

**Software and algorithms**		

MATLAB	MathWorks	https://www.mathworks.com/products/matlab.html
SigmaPlot	Grafiti	https://grafiti.com/
Graph theory analysis	Google	https://sites.google.com/site/bctnet/

### Experimental model and study participant details

#### Animals

Subjects (n = 3-5 brains per injection site; total subjects used = 35) were captive-bred sexually naïve male and female prairie voles descended from populations captured in southern Illinois. Voles were weaned on postnatal day 21 and housed with same-sex conspecific in cages (29.2 L x 19.1 W x 12.7 H cm) containing corn cob bedding and crinkle paper nesting material with food (LabDiet Rabbit Diet 5321) and water *ad libitum*. Colony rooms were maintained on a 12L:12D photoperiod (Lights on at 0600 h) and at a temperature range of 21 ± 1°C. Subjects were 70-150 days of age at the start of the experiment. All procedures were conducted in accordance with the National Institutes of Health Guide and Use of Laboratory Animals and the Institutional Animal Care and Use Committee and the University of Kansas.

### Method details

#### Stereotactic surgery

Voles were anesthetized with an i.p. injection of ketamine/dexmedetomidine cocktail (75/1 mg/kg) in sterile normal saline, then head-fixed into a stereotax (Stoelting). After leveling the head position using bregma and lambda as reference points, the skull was exposed via a small incision and holes were drilled to target each of the fifteen regions: ACC (n = 5; ML: ±0.48, DV: -2.17, AP: 1.34), AH (n = 4; ML: ±0.38, DV: -5.90, AP: -0.75), BLA (n = 4; ML: ±3.35, DV: -5.08, AP: -1.35), BNST (n = 3; ML: ±0.52, DV: -4.93, AP: -0.25, 5° angle), CA2 (n = 3; ML: ±2.56, DV: -2.01, AP: -2.18), CP (n = 5; ML: ±2.30, DV: -3.75, AP: 0.80), LS (n = 5; ML: ±0.62, DV: -3.90, AP: 0.80), MeA (n = 5; ML: ±3.01, DV: -5.83, AP: -1.30, 5° angle), mPOA (n = 4;ML: ±0.36, DV: -5.55, AP: -0.25), NAcc (n = 5; ML: ±1.27, DV: -5.63, AP: 1.40), PVN (n = 4; ML: ±0.19, DV: -5.20, AP: -0.74), PAG (n = 4; ML: ±0.53, DV: -3.50, AP: -3.28, 5° angle), VMH (n = 3; ML: ±0.38, DV: -6.20, AP: -1.45), VP (n = 5; ML: ±1.25, DV: -5.52, AP: 0.75), and VTA (n = 4; ML: ±0.79, DV: -4.91, AP: -3.20). All regions were injected into the right hemisphere.

Voles were randomly injected with 300 nl (either 60nl/min or 20nl/min) of each cholera toxin subunit-B retrograde tracer conjugated to either a 488nm (Invitrogen, Lot no. 2155272, Ref no. C34775), 555nm (Invitrogen, Lot no. 2129666, Ref no. C34776), 594nm (Invitrogen, Lot no. 2387433, Ref no. C34777), or 647nm (Invitrogen, Lot no. 2155574, Ref no. C34778) AlexaFluor, via a 1 μl syringe (Hamilton Syringe) into four regions. To ensure each CTB had similar cell labeling in the prairie vole, we injected a 300nl cocktail of each CTB (100nl each) into the nucleus accumbens shell and quantified 20X images of the ventral tegmental area for each CTB. Our cell counts for this validation step were: 184 (CTB-488), 183 (CTB-555), 185 (CTB-594), and 187 (CTB-647) (Figures S3A and S3B). Following the injection, the needle was left for an additional 5 minutes before slowly retracting it from the brain. The incision was closed using wound clips (Brantree Scientific Inc. 9mm, Cat no. 191840, lot no. 205016, Applicator no. 205000, Remover no. 205009).

#### Tissue preparation

Following 10 days of recovery, subjects were anesthetized with an i.p. injection of ketamine/dexmedetomidine cocktail (75/1 mg/kg) in sterile normal saline, then perfused through the ascending aorta with 15 ml of 0.9% saline, followed by 15 ml of 4% paraformaldehyde in 0.1 M phosphate-buffer (PB; pH 7.4). Brains were harvested, postfixed for 2 h in 4% paraformaldehyde at 4°C then stored in 30% sucrose in PB for 3 additional days. Next, brains were cut into 30 μm coronal sections, 180-μm apart, on a microtome (Leica SM2010 R: Sliding Microtome), which yielded approximately 24 pieces of tissue analyzed.

### Quantification and statistical analysis

#### Imaging quantification and analysis

All tissue was imaged on a Leica DM6-B Microscope with a 10x objective. Injection sites were histologically validated using the Allen Brain Atlas and validated that the retrograde CTB was contained within the regions. Brains with injections with partial hits or found outside of the target region were excluded. All quantified images can be found on the open-source repository DABI: https://doi.org/10.18120/t6yj-dg76. Whole-slice images were taken as a 6 x 9 grid and stitched together as one image. Cell counts of 30 μm sections collected at a 180-μm interval from rostral to caudal of a brain region were quantified by an experimenter. Thus, cell counting was performed on 1/6 or 16.67% of each brain region. If regions were highly innervated and the signal was too bright to quantify using the automated tool, these regions would be manually quantified. We developed modified brain templates, from the MRI Vole Brain Atlas (created by and generously given to us from Ferris, Kulkarni, and Kenkel), which used vectors to fit each brain slice appropriately and unique hexcodes assigned to each region to be analyzed by ImageJ.^108^ Each brain slice was appropriately assigned a template. This template was modified and adjusted to ensure proper fit for the brain slice. The brain slice and fitted template was imported into ImageJ, where we wrote an ImageJ Macro that would allow for quantification of each region associated to the imported template as well as quantify the ipsilateral and contralateral sides. Cell counts for ipsilateral and contralateral sides are in reference to the injection site, with ipsilateral being on the same hemisphere as the injection site. Furthermore, afferent cell counts refer to the number of cells that the injection site receives from a given region, and the efferent cell count is the retrograde labeled cells within the regions outside of the injection site. Our semi-automated quantification tool was validated to ensure a reliability of 99% when compared to manual quantification. This tool will be available upon request.

#### Statistics

For statistical analysis, three brains for each injected region were selected at random. Results for the difference between afferent and efferent contralateral and ipsilateral and total were obtained by performing an Unpaired T-Test. To assess differences between regional afferent/efferent total projections, we performed a One-Way Analysis of Variance (ANOVA) tests, with significant ANOVA results followed by Bonferroni post-hoc comparisons. Lastly, we performed a One-Way ANOVA for all regions quantified for each injected region. With significant results followed by a Bonferroni post-hoc comparison. Significant differences were determined by a p-value of <0.05∗.

#### Network analysis using graph theory

A graph theory approach was used to identify structural hubs of the SDMN network. Graph theory is a branch of mathematics that investigates patterns of connectivity between elements within a system, that represent graphs as nodes and edges. Graph theory is used a variety of disciplines such as organic chemistry, public transportation, finances, and other fields,^109^^,^^110^^,^^111^^,^^112^ as well as in neuroscience, particularly using graph theory to analyze topological organization of a system.^14^^,^^45^^,^^113^ Particularly in our case, nodes are represented by brain regions and edges are represented by physical projections. Hubs of a network are regions that are highly connected with other nodes or regions within the network.^114^ For the network analysis, an n of 3 brains for each target region were selected at random and were used for analysis. There are two types of hub analysis, unweighted hub analysis and a weight hub analysis.^13^^,^^30^^,^^45^ An unweighted hub analysis refers to the highest degree of connectivity based on the sum of edges present (1 edges = a count of 1). The contralateral and ipsilateral afferent and efferent projects of the regions in the vole social brain were added together to calculate the unweighted hub analysis. The second hub analysis performed was the weighted hub analysis. A weighted hub analysis assigns a particular weight to the edge present. In this present study, once the edges or physical projections were determined, each edge was assigned the quantified cell count for that particular projection. For an individual region total weight, the cell count of the ipsilateral and contralateral afferent and efferent projections for that region were summed. For the present study, the region with the highest strength of connectivity was proposed as a hub for this network.

In our network models we first generated dendrograms, using RStudio, based on the total afferent/efferent counts of each region and cut the dendrogram tree at a Euclidean height (vole social brain tree: 1100; Fore- and mid- brain tree: 1700) which tried to fit most regions within a module.^14^^,^^33^^,^^115^ Dendrograms identify which module a node or brain region will be in and regions that are part of the same module demonstrate similar structural connectivity patterns. We next assigned a ‘weight’ based on the cell count to generate the edges of the networks. The ‘weights’ are represented in Table S1.

The weight is displayed in the networks based on the size of the arrow, which also provides the directionality of the projection (Figure 9). To further characterize individual nodes of this network, we first calculated a within-module degree z-score (WMDz) to determine the connectivity a node has within its own model. The WMDz for node *I*, Equation 1, is defined as:

(Equation 1) $$WMDz=\frac{K_{i-\bar{K}s_{i}}}{{\sigma_{s}}_{i}}$$

$k_{i}$ is the sum of the weights of the edges that node *i* has to other nodes in the same module, *s*_*i*_ is the total number of nodes within module *s*, and ${\bar{K}}_{s_{i}}$ is the average of k over all the nodes $s_{i}$, and ${\sigma_{s}}_{i}$ is the standard deviation of k in $s_{i}$. If a node is in a module by itself, that node has a WMDz of 0 by default.

Lastly, the participation coefficient (PC) was calculated to assess the distribution of edges between different modules.^57^ The PC has a range of 0 to 1 with 0 meaning no participation outside its own module and approaching 1 reflecting the greatest participation with different modules. For the participation coefficient, Equation 2, *K*_*is*_ (between-module degree) is the sum of the weights of edges from node *i* to nodes in module *s. k*_*i*_ (total degree) is the total strength of node *i*, which is the sum of the weights of all edges connected to node *i*. The participation coefficient of each node, PC*,* is then defined as:

(Equation 2) $$PC=1-\sum_{s=1}^{N_{M}}{\left(\frac{K_{is}}{k_{i}}\right)}^{2}$$

We used Python to calculate the WMDz and PC and used Gephi 0.10 software package to visualize our structural connectivity network (Brain Connectivity Toolbox).^116^

#### Cartography of the network

Cartographic models were generated using RStudio and the *scatterplot3d* package. Once the within-module degree z-score (WMDz) and participation coefficient (PC) were calculated as described above, the PC was plotted on the x-axis, the degree of strength or total cell count from the weighted hub analysis was plotted on the y-axis, and the WMDz was plotted on the z-axis. We followed criteria established by Guimera and Amara (2005) for defining nodes’ organizational roles.^32^ Peripheral nodes were defined as nodes that have at least 60% of its links within its own module, the *k* < 4 it follows that *P* < 0.625. Non-hub connectors were nodes with *k* < 4 and half of its links within its own module, follows 0.62 < *P* < 0.8.
