# Supplementary material for: Structural connectivity of the fore- and mid-brain in prairie voles
Source: iScience. 2025 Feb 20;28(3):112065. doi: 10.1016/j.isci.2025.112065 (PMC11938270; doi:10.1016/j.isci.2025.112065)
Supplement: Document S1. Figures S1–S3 and Table S1 [file mmc1.pdf]

iScience, Volume *28*

Supplemental information

Structural connectivity

# of the fore- and mid-brain in prairie voles

Kyle R. Gossman, Emalee Andrews, Ben Dykstra, Kyle Ta, Arian Ashourvan, and Adam S. Smith

A

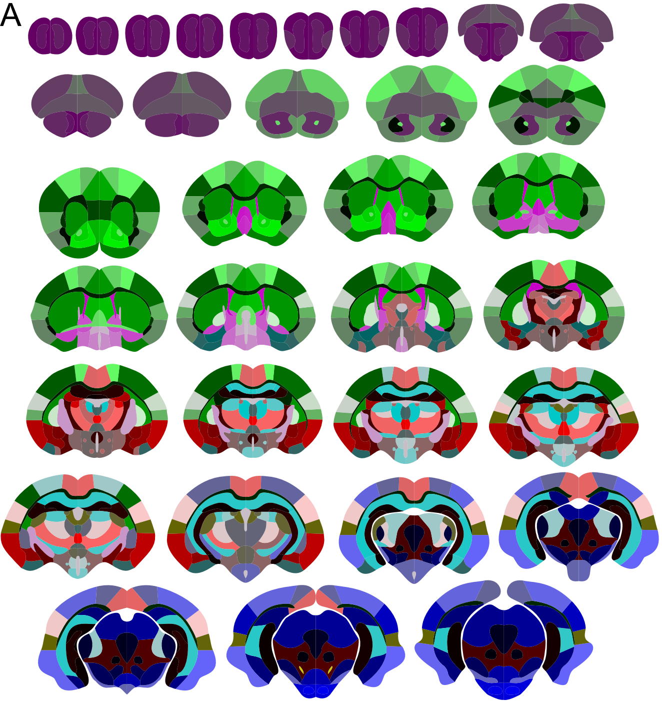

B

Region	Abbreviation		
Agranular insular cortex	AI	Medial dorsal thalamic nucleus	MD
Anterior amygdaloid nucleus	AA	Medial geniculate	MG
Anterior cingulate cortex	ACC	Medial preoptic area	mPOA
Anterior hypothalmic area	AH	Medial septum	MS
Anterior olfactory nucleus	AO	Nucleus accumbens core	NAc
Anterior thalamic nuclei	A	Nucleus accumbens shell	NAs
Auditory cortex	Au	Nucleus lateral olfactory tract	LOT
Basal amygdaloid nucleus	BLA	Olfactory tubercles	Tu
Bed nucleus of the stria terminalis	BNST	Orbital cortex	OFC
CA1	CA1	Parafascicular thalamic cucleus	PF
CA2	CA2	Paraventricular hypothalamic nucleus	PVN
CA3	CA3	Paraventricular thalamic nucleus	PVA
Caudal piriform cortex	PirC	Parietal cortex	Pt
Caudate putamen (Striatum)	CP	Periaqueductal gray	PAG
Central amygdaloid nucleus	Ce	Perirhinal cortex	PRh
Central medial thalamic nucleus	CM	Posterior hypothalamic area	PH
Claustrum	Cl	Posterior thalamic nucleus	PO
Cortical Amygdaloid Nucleus	Co	Prelimbic cortex	PrL
Dentate gyrus	DG	Primary motor cortex	M1
Diagonal band of Brcoa	DB	Primary somatosensory cortex	S1
Dorsal medial nucleus	DM	Red nucleus	R
Endopiriform nucleus	EN	Reticular nucleus	Rt
Entorhinal cortex	Ent	Retrosplenial cortex	RS
Extended amygdala	EA	Reuniens nucleus	Re
Frontal association cortex	FrA	Rostral piriform cortex	PirR
Globus Pallidus	GP	Secondary Somatosensory	S2
Granule cell layer of the olfactory bulb	Gr	Secondary motor cortex	M2
Habenula nucleus	Hb	Strial part of the preoptic area	StA
Inferior Colliculus	IC	Subiculum	S
Infralimbic cortex	IL	Substantia nigra	SN
Interpeduncular nucleus	IP	Superior colliculus	SC
Lateral amygdaloid nucleus	La	Temporal Cortex	Te
Lateral dorsal thalamic nucleus	LD	Tenia tecta cortex	TT
Lateral geniculate	LG	Ventral medial nucleus	VMH
Lateral hypothalamus	LH	Ventral pallidum	VP
Lateral posterior thalamic nucleus	LP	Ventral tegmental area	VTA
Lateral preoptic area	LPO	Ventral thalamic nuclei	V
Lateral septal nucleus	LS	Visual 1 cortex	V1
Mammillary nucleus	MM	Visual 2 cortex	V2
Medial amygdaloid nucleus	MeA	Zona incerta	ZI

Figure S1. Representative templates created from the vole fMRI and the associated hex-colors (A). All regions quantified along with the abbreviations for each region (B).

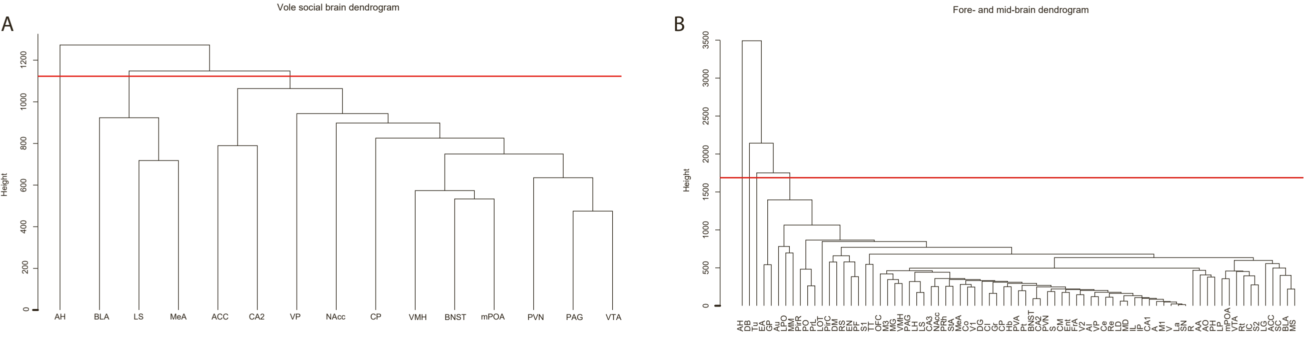

Figure S2. Hierarchical clustering or dendrograms were generated to assess similar projection patterns between two or more regions. Specifically, the Euclidean distance was used to generate the dendrogram for the vole social brain (tree cut at 1100) (A) and all regions (tree cut at 1700) (B). The red line demonstrates where the dendrograms were cut to determine the clusters or modules for the networks.

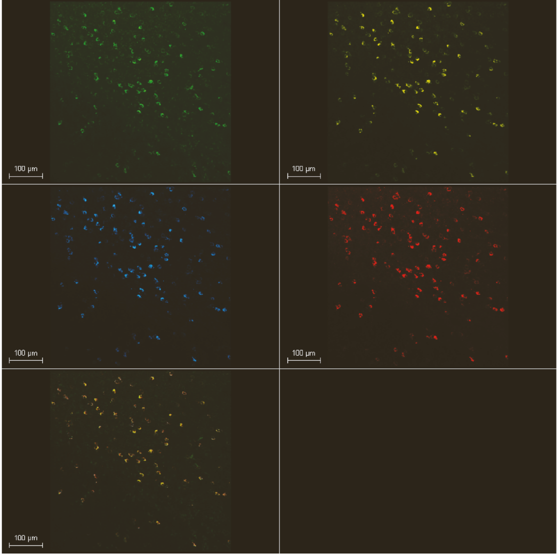

Channel/CTB	CTB-488	CTB-555	CTB-594	CTB-647	GYBR
Cell counts	184	183	185	187	170

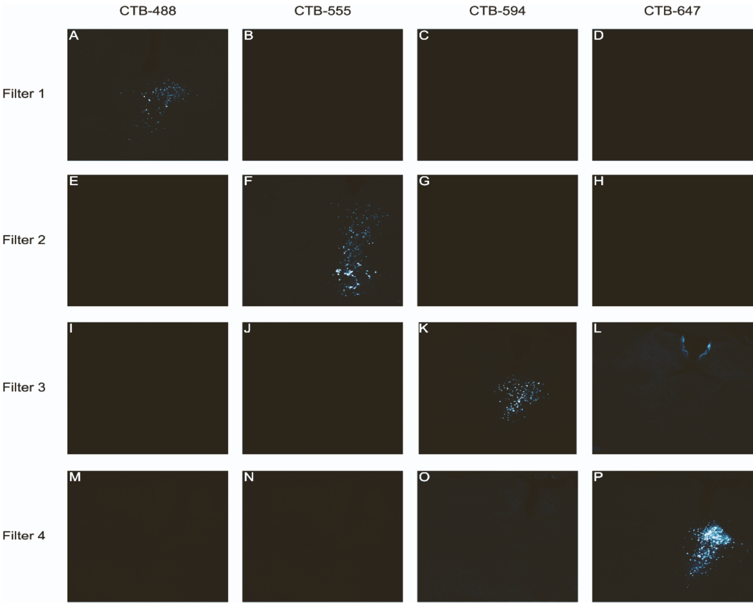

Figure S3. Methods validation of: Cholera toxin subunit-B retrograde tracers (A) (CTB-488: row 1 column 1, CTB-555: row 1 column 2, CTB-594 (pseudo-colored blue): row 2 column 1, CTB-647: row 2 column 2, merged image: row 3 column 1) and filter validation (B) for each CTB. Counts of each CTB are represented in the table in A. Scale bar = 100 µm.

Table S1. Edge weights of the network determined by cell counts

Cell Count	Edge Weight
0	0
1-20	0.1
21-40	0.2
41-60	0.3
61-80	0.4
81-100	0.5
101-150	0.6
151-200	0.7
201-250	0.8
250+	0.9
